# Genome-Wide Association Study of Zinc Toxicity Tolerance within a Rice Core Collection (*Oryza sativa* L.)

**DOI:** 10.3390/plants11223138

**Published:** 2022-11-16

**Authors:** Kaizhen Zhong, Lihong Xie, Shikai Hu, Gaoneng Shao, Zhonghua Sheng, Guiai Jiao, Ling Wang, Ying Chen, Shaoqing Tang, Xiangjin Wei, Peng Zhang, Peisong Hu

**Affiliations:** 1 College of Agronomy, Jiangxi Agricultural University, Nanchang 330045, China; 2 State Key Laboratory of Rice Biology, China National Rice Research Institute, Hangzhou 310006, China

**Keywords:** rice, Ting’s core collection, Zn toxicity, GWAS, QTL

## Abstract

Zinc (Zn) is an essential micronutrient for rice, but it is toxic at a high concentration, especially in acid soils. It is yet unknown which genes regulate Zn tolerance in rice. In the present study, a genome-wide association study (GWAS) was performed for Zn tolerance in rice at the seedling stage within a rice core collection, named Ting’s core collection, which showed extensive phenotypic variations in Zn toxicity with high-density single-nucleotide polymorphisms (SNPs). A total of 7 and 19 quantitative trait loci (QTL) were detected using root elongation (RE) and relative root elongation (RRE) under high Zn toxicity, respectively. Among them, 24 QTL were novel, and *qRRE15* was located in the same region where 3 QTL were reported previously. In addition, *qRE4* and *qRRE9* were identical. Furthermore, we found eight candidate genes that are involved in abiotic and biotic stress, immunity, cell expansion, and phosphate transport in the loci of *qRRE8*, *qRRE9*, and *qRRE15*. Moreover, four candidate genes, i.e., *Os01g0200700*, *Os06g0621900*, *Os06g0493600*, and *Os06g0622700*, were verified correlating to Zn tolerance in rice by quantitative real time-PCR (qRT-PCR). Taken together, these results provide significant insight into the genetic basis for Zn toxicity tolerance and tolerant germplasm for developing rice tolerance to Zn toxicity and improving rice production in Zn-contaminated soils.

## 1. Introduction

Zinc (Zn) is an essential micronutrient for rice growth and development, as it is involved in numerous physiological and biochemical processes [1]. However, human activities, such as mining, sewage sludge treatment in agricultural soils, and anthropogenic Zn inputs in urban and peri-urban soils, cause Zn contamination [1,2]. Excess Zn in soil is toxic to rice and results in growth inhibition, as well as yield reduction, especially in acidic soils [1]. Therefore, improving Zn tolerance is significantly meaningful to rice production.

Deciphering the genetic mechanism for Zn tolerance in rice is the premise of improving rice Zn tolerance. Plants have developed resistant mechanisms responding to Zn toxicity, which include translocation from root to shoot, sequestration into vacuoles, and homeostasis with iron [3,4,5,6,7,8,9,10,11]. Moreover, rice show a wide range of natural variations and differ markedly in their susceptibility to Zn toxicity [12,13,14,15,16]. Plant physiologists and breeders have been focusing on revealing the genetic mechanism of Zn tolerance in rice; however, more studies still need to be performed, in the future, due to the complexity of the genetic mechanism for Zn tolerance in rice.

A total of 74 QTL for Zn tolerance in rice have been reported in previous research via linkage mapping and GWAS [12,14,15,16]. These QTL are associated with some easily measurable traits, such as leaf discoloring index, shoot height, root length, and shoot and root fresh weight and dry weight under Zn toxicity conditions at the seedling stage. For instance, a major QTL, *qZNT-1* on chromosome 1, shows an effect with an LOD value of 6.0 and explains 21.9% of the total phenotypic variation using leaf discoloring index [12]. *qZnRL11* from chromosome 11 [15] is co-located with the QTL for relative shoot dry weight and relative total dry weight [14]. *qSFW3b* for shoot fresh weight and *qSDW3b* for shoot dry weight [16] are co-located with *qZNT-3* [12]. We summarized QTL and found that rice Zn tolerance genes were not cloned, and this was, for the greater part, due to the difficulty of evaluating Zn tolerance in rice.

Phenotyping is a prerequisite for evaluating heavy metal tolerance in plants. In *Arabidopsis*, root length growing under heavy metal treatment is frequently used to evaluate heavy metal tolerance, and some tolerant and sensitive accessions were identified by using root length under heavy metal toxicity. Furthermore, well-known genes such as *FRO2*, *GSNOR*, and *SRF3* were located through GWAS [17,18,19,20].

A variety of plants from highly contrasting environments showed extensive natural variation and increased the probability of identifying causal variants [20]. Rice landraces from Ting’s core collection were collected from different environments [21], which showed abundant genetic diversity and significantly different adaptations to abiotic stress, especially cadmium toxicity (data not provided) and aluminum toxicity [22,23]. Ting’s core collection was used for GWAS on rice agronomic traits [24], aluminum tolerance [22,23], and sheath blight resistance [25]. Therefore, Ting’s core collection might be an appropriate population for GWAS on rice Zn tolerance.

In the present study, we performed a GWAS with more than 4.3 million high-quality SNPs by using root elongation and relative root elongation (root elongation in high Zn conditions/root elongation in control conditions) under high Zn toxicity at the seedling stage within Ting’s core collection. This study aims to (1) identify the candidate genes corresponding to Zn toxicity and (2) evaluate the landraces with strong Zn tolerance in Ting’s core collection. This study may provide information for improving rice Zn tolerance in potentially Zn-contaminated soils.

## 2. Results

### 2.1. Identification of Phenotypic Variations for Zn Tolerance within Ting’s Core Collection

**Figure 1:**
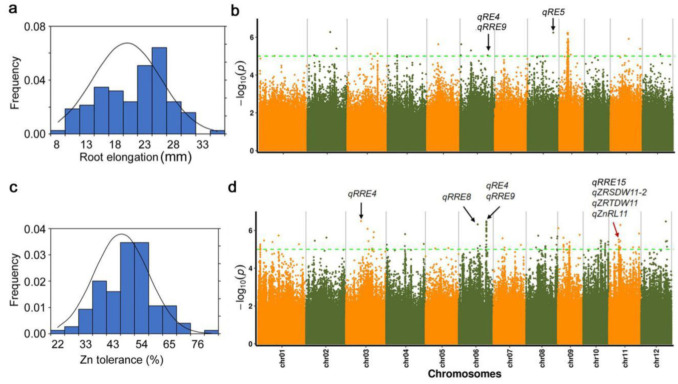
GWAS of Zn tolerance by using EMMAX model. (**a**) Distribution of root elongation of Ting’s core collection growing under Zn toxicity (120 μM); (**b**) Manhattan plot of GWAS using root elongation under Zn toxicity (120 μM); (**c**) distribution of relative root elongation of Ting’s core collection growing under Zn toxicity (120 μM); and (**d**) Manhattan plot of GWAS using relative root elongation. Chromosomes are depicted in different colors. The horizontal green dashed line indicates the significance threshold (−log10(mBF) = 5.02). The black arrows represent the novel loci associated with Zn tolerance detected in the present study. The red arrow represents the loci, which were reported previously.

We made use of Ting’s core collection and assessed the phenotypic variations in root elongation under Zn toxicity. The root elongation (RE) and relative root elongation (RRE, root elongation in high Zn/root elongation in control) of Ting’s core collection were measured before and after Zn exposure. Extensive phenotypic variations in root elongation in response to Zn toxicity were observed (Figure 1a,c; Table S1). The average root elongation was 43.54 mm with a range from 16.71 mm to 56.12 mm under control conditions and 20.16 mm with a range of 8.25–34.39 mm under high Zn conditions. The average relative root elongation was 46.42%, ranging from 21.86% to 78.19% (Table 1). On the basis of the value of relative root elongation, one variety, named You zhan hong, which was collected from South China, was inhibited under high Zn toxicity (relative root elongation was 21.86%), while one variety, named Nagabo, from Taiwan of China, showed the highest Zn tolerance (relative root elongation was 78.19%) (Table S1).

### 2.2. GWAS for Root Elongation under High Zn Toxicity within Ting’s Core Collection

The data of genetic analysis referenced from our previous studies, in which genetic analysis included the linkage disequilibrium (LD) decay distance, population structure, and kinship value of Ting’s core collection required for GWAS, were uncovered well.

A total of 4,395,003 high-quality SNPs were used to perform GWAS. In the present study, seven novel QTL on chromosomes 2, 6, 8, 9, and 11 were identified as significantly (*p* < 0.94 × 10^−5^) associated with root elongation under high Zn toxicity by using the EMMAX model (Table 2). Among them, *qRE4* and *qRE5* were still detected by using the LMM model (Figure 1b). While there were 37 QTL detected as associated with root elongation without Zn toxicity, the same QTL were not found with root elongation under high Zn toxicity and without Zn toxicity (Table 2 and Table S2).

**Figure 2:**
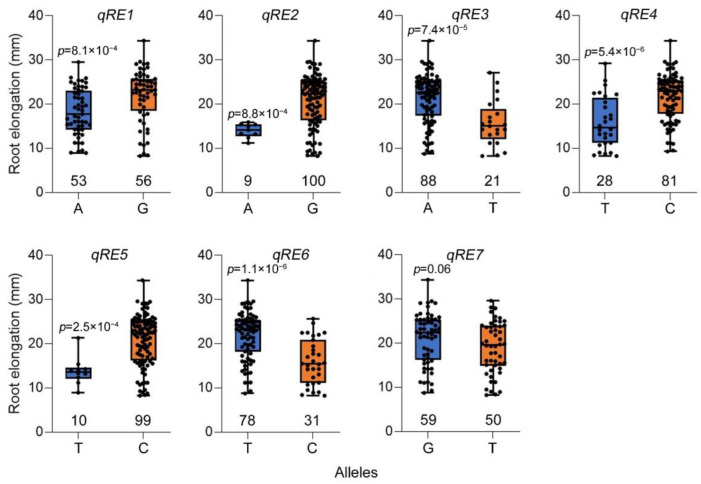
Effect analysis of allelic variations on root elongation under high Zn toxicity.

Additionally, we focused on QTL associated with root elongation under high Zn conditions. The most significant SNP for each QTL was selected to detect the effects of allelic variations on root elongation under high Zn toxicity (Figure 2 and Table 2). Under high Zn toxicity, the root elongation of varieties with reference (Nipponbare) alleles was significantly larger than those with alternative alleles in Ting’s core collection, whereby the most significant SNPs of *qRE1*, *qRE2*, *qRE4*, and *qRE5*. However, the root elongation of varieties with reference (Nipponbare) alleles was significantly smaller than those with alternative alleles in Ting’s core collection, whereby the most important SNPs of *qRE3* and *qRE6* (Figure 2).

### 2.3. GWAS for Relative Root Elongation under High Zn Toxicity within Ting’s Core Collection

We then focused on the QTL associated with relative root elongation. In the present study, 19 novel QTL distributed on all chromosomes, except chr05, were identified as significantly (*p* < 0.94 × 10^−5^) associated with root elongation under high Zn toxicity by using the EMMAX model (Table 3). Among them, *qRRE4*, *qRRE8* and *qRRE9* were detected by using the LMM model (Figure 1d). Furthermore, there were two QTL, i.e., *qRE4* and *qRRE9*, located in the same region on chromosome 6 (Figure 1b, d). Moreover, *qRRE1* was located in the region of *OsMTI-3a,* which was reported to relate to Zn tolerance in yeast (Table 3), and *qRRE15* was identical to three previous QTL, i.e., *qZRSDW11-2*, *qZRTDW11*, and *qZnRL11* (Figure 1d).

**Figure 3:**
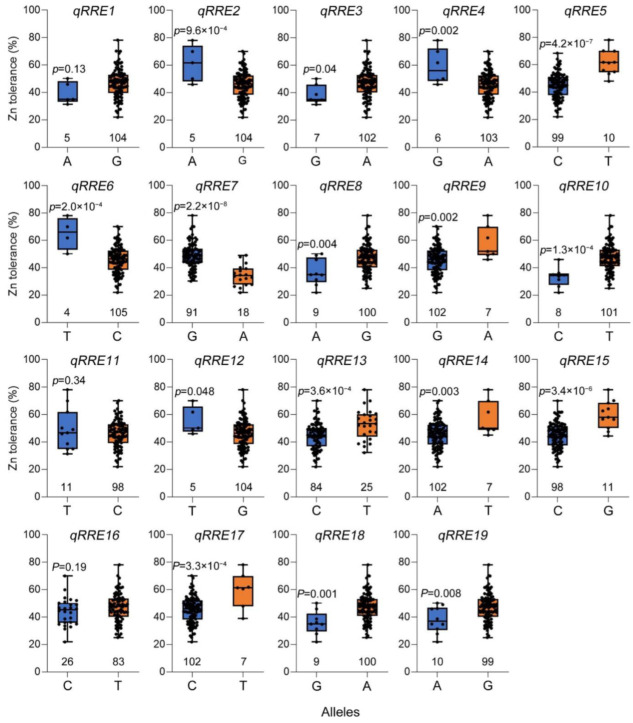
Effect analysis of allelic variations on relative root elongation under high Zn toxicity.

The most significant SNP for each QTL was selected for detecting the effects of allelic variations on relative root elongation under high Zn toxicity (Figure 3 and Table 3). Under high Zn toxicity, the varieties with reference (Nipponbare) alleles were significantly more tolerant than those with alternative alleles, which were grouped by the most significant SNPs of *qREE2*, *qRRE4*, *qRRE6*, *qRRE7*, and *qRRE12*. However, the varieties with reference (Nipponbare) alleles were significantly more sensitive than those with alternative alleles in Ting’s core collection, which were grouped by the most significant SNPs of *qRRE3*, *qRRE5*, *qRRE8*, *qRRE9*, *qRRE10*, *qRRE13*, *qRRE14*, *qRRE15*, *qRRE17*, *qRRE18*, and *qRRE19* (Figure 3).

### 2.4. Candidate Genes Analysis of Detected QTL Responding to Zn Toxicity in the Present Study

After detecting QTL associated with Zn toxicity tolerance, we sought to isolate the genes that regulate Zn tolerance in rice. We analyzed the candidate genes of QTL (Table S3) according to the following reasons: firstly, we chose QTL detected using both the EMMAX and LMM models, and *qRE5*, *qRE4*/*qRRE9*, *qRRE4* as well as *qRRE8* were detected both in EMMAX and LMM; secondly, we selected QTL that were identical to previous QTL, i.e., *qRRE15*; and thirdly, the genes in Fe homeostasis, Zn chelator, phosphate transporters, and MATE efflux families have been shown to be related to Zn tolerance, and we found that there were many genes in the above families annotated in the regions of *qRE2*, *qRRE1*, and *qRRE12*.

**Figure 4:**
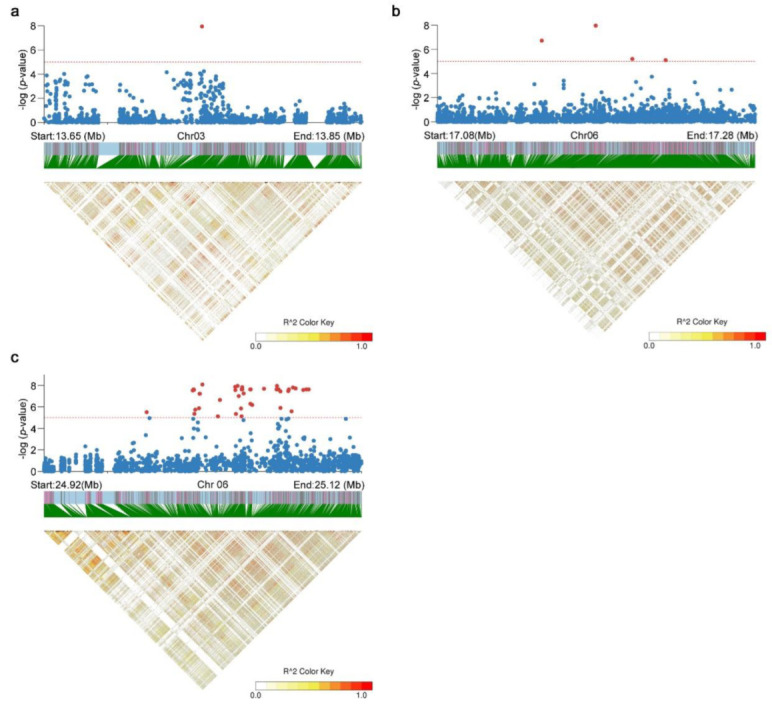
Local Manhattan plot (top panel) and LD heatmap (bottom panel) of the 13.65–13.85 Mb region of chromosome 3 (**a**), 17.08–17.28 Mb region of chromosome 6 (**b**), and 24.91–25.11 Mb region of chromosome 6 (**c**).

On the basis of the above reasons, the information about candidate genes is detailed as follows: For *qRE2* on chromosome 2, there were 25 candidate genes in the region 26.20–26.39 Mb. Among these candidate genes, one gene (*Os02g0650300*) was an iron (III)–deoxymugineic acid transporter (*OsYSL15*), which is responsible for iron uptake and the phloem transport of iron [26,27]. For *qRRE1* on chromosome 1, there were 29 candidate genes in the region 5.38–5.59 Mb. One candidate was *Os01g0200700* (*OsMTI-3a*), which is involved in the detoxification of Zn, Ni, and Cd [28]. For *qRRE4* on chromosome 3, there was only one significant SNP and 17 candidate genes in the region 13.65–13.85 Mb (Figure 4a). For *qRRE8* on chromosome 6, there were 15 candidate genes in the region 17.13–17.29 Mb, which were covered by four significant SNPs (Figure 4b). One candidate was a phosphate transporter (OsPHO1;3), and two candidates were from the MATE efflux family. The most significant peak (*qRRE9*) of all these loci was located on chromosome 6 around the top SNP at position 25,053,430. The candidate region was from 24.92 Mb to 25.12 Mb (200 kb) by LD calculation and contained twenty-two candidate genes, which were covered by 35 significantly associated SNPs (Figure 4c). For *qRRE12*, there were 26 candidate genes in the region 28.14–28.34 Mb. One candidate gene was *Os08g0564000* (*OsPT6*), which plays a role in the uptake and the long-distance transport of Pi from roots to shoots, [29] and another one was a MATE efflux family protein. For *qRRE15*, there were sixteen candidate genes in the region 8.88–9.08 Mb, which were covered by eight significant SNPs.

**Figure 5:**
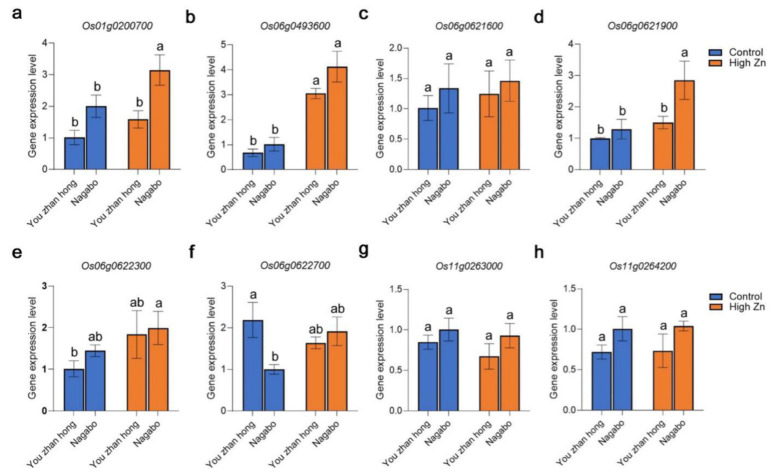
The gene expression of 8 candidate genes in representative varieties (the most sensitive variety in the present study, You zhan hong; the most tolerant variety in the present study, Nagabo) in control and high Zn conditions as measured by qRT-PCR. Error bars: S.D. The different letters indicate significant differences by one-way ANOVA analysis with Tukey’s HSD test (*p* < 0.05, n = 3).

Furthermore, eight candidate genes detected by GWAS were selected to verify by qRT-PCR. Among the eight candidate genes, the expression of three genes was significantly different in the roots between Nagabo (Zn tolerant) and You zhan hong (Zn sensitive) (Figure 5a,d,f) under Zn toxicity. While the expression of the gene in Figure 5b was induced by Zn toxicity, the expression of the remaining four candidate genes was not significant between Zn-tolerant and Zn-sensitive varieties, nor was it induced by Zn toxicity (Figure 5c,e,g,h).

## 3. Discussion

Ting’s core collection may be a potential resource of elite genes/alleles for Zn tolerance in rice due to abundant and useful natural variations for agronomic traits, aluminum tolerance, as well as rice sheath blight resistance, which have been reported within this core collection in our previous studies [22,23,24,25]. In fact, the extension of phenotypic variations for Zn tolerance within Ting’s core collection is large in the present study (Figure 1a,c, Table 1). Moreover, rice varieties with strong Zn tolerance were discovered in the present study (Table S1).

The concentrations of Zn toxicity used in previous studies were diverse due to different populations and methods, e.g., 300 mg/L and 200 mg/L were used in the studies of Meng et al. (2017) [15] and Zhang et al. (2017) [16]. Moreover, we do not think that positive QTL regarding Zn tolerance will be identified by using an unsuitable Zn toxicity concentration. Thus, several concentrations (50 μM, 120 μM, 150 μM, 200 μM, and 300 μM, data not shown) of Zn toxicity were used to determine suitable treatment concentration, and a concentration of 120 μM was distinct for Zn tolerance among Ting’s core collection. Furthermore, the concentration was proven suitable, from the results of this GWAS, because identical QTL were not found in root elongation under high Zn toxicity and without Zn toxicity (Table 2 and Table S2). The above evidence supports the results of this GWAS.

Furthermore, a number of novel QTL, which are identical to previous QTL, and previously identified QTL were uncovered in this GWAS. A total of 4,395,003 high-quality SNPs were used to perform this GWAS using the EMMAX and LMM models. The above super-density SNPs covered most of the genomic regions for Ting’s core collection; thus, the results of the GWAS in the present study may offer reliability and accuracy in LD decay distance, population structure, and kinship value, which should have been the requirements of the GWAS in our previous studies [22,23,24,25]. Indeed, we can only speculate facts based on the above-mentioned information. In the present study, we found that all significant QTL identified by EMMAX were also detected by LMM, and three QTL (*qRRE4*, *qRRE8*, and *qRRE9*) using relative root elongation under Zn toxicity exceeded the supposedly stringent significance threshold (−log10(1/n) = 7.64, Figure S2b) when the LMM model was used. For *qRRE4* and *qRRE8*, there were only one and four SNPs, respectively, within a candidate region of 200 kb distance surrounding the most significant SNP. For *qRRE9*, there were thirty-five SNPs surrounding the most significant SNP at position 25,053,430 on chromosome 6 (Figure 4c). We speculate that it is worthwhile to dedicate more works on *qRRE9* in the future because obvious LD decay was uncovered in this QTL (Figure 4c).

Furthermore, in the present study, the same QTL were detected by using different phenotypes, as well as identified in previous studies. When comparing QTL detected by root elongation and relative root elongation under high Zn toxicity, we found that *qRE4* was located in the same region as *qRRE9* (Figure 1). When comparing QTL identified in the present study (Table 2 and Table 3) with previously reported Zn-tolerant QTL, we found that *qRRE15*, which is located on chromosome 11, was mapped in the same region as QTL (*qZRSDW11-2* and *qZRTDW11*) detected by relative shoot dry weight and relative total dry weight using reciprocal introgression populations [14] and a QTL (*qZnRL11*) detected by root length using MAGIC populations [15]. These results suggest that *qRE4*/*qRRE9* and *qRRE15* may be the causal loci involved in Zn toxicity tolerance.

Although many QTL associated with Zn toxicity tolerance in rice have been detected using different traits [12,14,15,16], the major quantitative genetic variants have not yet been fine-mapped or cloned, which means little is known about the genetic mechanisms of rice tolerant to Zn toxicity. Using high-density SNPs and natural variations in root elongation for GWAS analysis, we detected a number of novel QTL.

There is already vast knowledge on the molecular mechanisms of Zn homeostasis in plants, which was summarized in a review of Kaur and Garg (2021) [30]. Previous studies have shown that excess Zn caused Fe deficiency-induced chlorosis through chlorophyll synthesis reduction, chloroplast degradation, and reduced P levels in shoots [2]. Moreover, *AtFRD3*, which encodes a member of the MATE efflux family, has been demonstrated to play a role in cross homeostasis between Fe and Zn tolerance in *Arabidopsis* [11]. Yellow stripe 1-like (YSL) genes encode transporters, which can transport iron, Zn, and nickel NA complexes [31]. Transgenic plants and yeasts that carry *OsMT1a* accumulate more Zn than wild type [32]. Thus, we further analyzed the candidate genes in the region of some loci, which included the genes in Fe homeostasis, Zn chelator, phosphate transporters, and the MATE efflux family (Table S3). In the present study, we detected *OsYSL15* located in the region of *qRE2*, *OsMTI-3a* in the region of *qRRE1*, *OsPHO1;3* and two MATE efflux-related genes in the region of *qRRE8*, and *OsPT6*, as well as a MATE efflux gene, in the region of *qRRE12*. In addition, qRT-PCR was performed to support the candidate genes identified in the present study; we will deeply study four candidate genes, i.e., *Os01g0200700*, *Os06g0621900*, *Os06g0493600*, and *Os06g0622700*, by using technology such as CRISPR-Cas9 and over-expression. Therefore, the above results suggest that these genes might be the causal genes that respond to Zn toxicity. However, the molecular mechanism of tolerance under Zn toxicity is complicated; hence, further studies are necessary to confirm the candidate genes and reveal the molecular mechanisms of rice adaptation to Zn toxicity.

Moreover, Zn-tolerant (Nagabo, Duan mang zi jin gu, and Bu gou wei) and Zn-sensitive varieties (You zhan hong) are suitable genetic resources for improving the Zn tolerance of rice cultivars. Therefore, the tolerant elite alleles in these varieties can be pyramided by marker-assisted breeding or edited directly by CRISPR-Cas9 in an elite, modern cultivar background.

## 4. Materials and Methods

### 4.1. Plant Materials

Ting’s core collection, which consisted of 150 rice landraces, was collected from China, Korea, Japan, the Philippines, Java, Oceania, Vietnam, and Brazil [21] (Table S1).

### 4.2. Phenotyping for Zn Toxicity Tolerance

The seeds of Ting’s core collection were harvested from the field of China National Rice Research Institute, Hangzhou in 2020. The seeds were soaked in water for 2 days at 30 °C in the dark, transferred to a 1 L pot with 96-well PCR plates (8 × 12) in a solution containing 0.5 mM CaCl_2_ (pH = 5.6). After 1 day, the seedlings were subjected to a solution of 0.5 mM CaCl_2_ containing 0 (control) and 120 μM ZnSO_4_ for 24 h. The experiment was conducted using a randomized complete block design with three replicates and 6 uniformly germinated seeds. The root length (RE) was measured by a ruler before and after the Zn treatment. Relative root elongation (RRE) was calculated based on (root elongation in high Zn/ root elongation in control conditions) × 100 [33]. The screening for Zn was conducted in a growth chamber with day/night temperature of 30/28 °C and relative humidity of 75%.

### 4.3. SNPs Calling

The 4,395,003 SNPs used for GWAS were based on re-sequencing performed by Illumina HiSeq^TM^ 4000 with 6~7-fold of genome coverage; detailed information was described in our previous study [22,24]. The SNPs with low minor allele frequency (0.05) were removed, and, finally, 4,393,003 SNPs were selected for our GWAS analysis.

### 4.4. GWAS Analysis

We performed GWAS to detect the trait–SNP associations for Zn tolerance using 4,395,003 SNPs and the mean value and tolerance index of root elongation at high Zn. Two mixed linear models were used, the EMMAX and LMM methods. The critical *p*-value threshold for identifying the significant marker–trait associations in our study was 2.27 × 10^−7^ (*p* = 1/n; n = total markers), which is a rough Bonferroni correction corresponding to −log10(*p*) = 7.64. However, when there was no peak detected, another significance threshold was calculated, i.e., a minimum Bayes factor (mBF). The mBF was calculated using the following formula: mBF = −e*p*ln(*p*) [22], thus, the significance threshold in present study was -log10(mBF) = 5.02. Moreover, the peaks exhibiting a significance threshold level within a physical distance of about 200 kb were merged into a single QTL.

### 4.5. qRT-PCR

The seedlings were grown in 0.5 mM CaCl_2_ solution (pH = 5.6) for 1 day after germination, and then subjected to a 0.5 mM CaCl_2_ solution containing 0 μM (control) and 120 μM (high Zn) Zn concentrations for 12 h. Whole roots were harvested and immediately frozen in liquid nitrogen. Total RNA was extracted using the FastPure Universal Plant Total RNA Isolation Kit (Vazyme, Nanjing, China). First-strand cDNA was synthesized using the ABScrip III RT Master Mix (ABclonal, Wuhang, China). qRT-PCR reaction was prepared using the THUNDERBIRD SYBR qPCR Mix (TOYOBO, Tokyo, Japan). Three biological replicates and two technical replicates were analyzed for each gene. Relative expression of all genes was normalized using *OsUBQ1* (*Os03g0234350*) as an internal reference. The primers used for qRT-PCR are shown in Table S4.

## 5. Conclusions

In the present study, a total of 7 and 19 QTL were identified as significantly associated with Zn tolerance using root elongation (RE) and relative root elongation (RRE) under high Zn toxicity. Among them, *qRE4*, which was detected at high Zn concentrations, was located in the same region as *qRRE9*. *qRRE15* was located in the same region of three previously reported QTL (*qZnRL11*, *qZRSDW11-2*, and *qZRTDW11*). These results indicate that there may be causal genes regulating Zn tolerance in the above-mentioned QTL. Furthermore, a number of candidate genes were detected in QTL in the present study, including the genes involved in Fe homeostasis, metal chelators, and Pi transporters. Moreover, eight candidate genes were verified through qRT-PCR. Three varieties (Nagabo, Duan mang zi jin gu, and Bu gou wei) had elite alleles identified in the QTL associated with high Zn tolerance. Taken together, our findings provide significant insight into the genetic basis for Zn toxicity tolerance and will be very useful for developing rice varieties that are tolerant to Zn toxicity and improving rice production in Zn-contaminated soils.

## Figures and Tables

**Table 1:** Performance of Ting’s core collection under control conditions and Zn toxicity.

Trait	Range	Mean ± SD
Root elongation (RE, control)	16.71–56.12 (mm)	43.54 ± 8.95 (mm)
Root elongation (RE, under 120 μM Zn)	8.25–34.39 (mm)	20.16 ± 5.91 (mm)
Relative root elongation (RRE, %)	21.86–78.19 (%)	46.42 ± 10.48 (%)

**Table 2:** Novel QTL for root elongation under high Zn toxicity in Ting’s core collection.

QTL	Chromosome	Position	*p*-Value	Allele (the Reference/the Alternative)
*qRE1*	chr02	20,681,680	5.34 × 10^−7^	A/G
*qRE2*	chr02	26,288,978	3.98 × 10^−6^	A/G
*qRE3*	chr06	1,118,739	2.35 × 10^−6^	A/T
*qRE4*	chr06	25,006,144	9.09 × 10^−6^	T/C
*qRE5*	chr08	23,227,944	5.70 × 10^−7^	T/C
*qRE6*	chr09	7,341,101	8.07 × 10^−8^	T/C
*qRE7*	chr11	27,019,946	4.12 × 10^−6^	G/T

RE: root elongation.

**Table 3:** QTL for relative root elongation identified in Ting’s core collection.

QTL	Chromosome	Position	*p*-Value	Allele (the Reference/the Alternative)	Previously Reported QTL
*qRRE1*	chr01	5,483,287	3.43 × 10^−6^	A/G	*OsMTI-3a* [28]
*qRRE2*	chr01	18,578,810	1.84 × 10^−6^	A/G	
*qRRE3*	chr02	7,969,392	3.50 × 10^−6^	G/A	
*qRRE4*	chr03	13,749,414	3.16 × 10^−7^	G/A	
*qRRE5*	chr03	19,713,560	8.19 × 10^−7^	C/T	
*qRRE6*	chr03	25,346,195	1.22 × 10^−6^	T/C	
*qRRE7*	chr04	17,157,142	1.56 × 10^−6^	G/A	
*qRRE8*	chr06	17,185,339	4.76 × 10^−7^	A/G	
*qRRE9*	chr06	25,053,430	3.29 × 10^−7^	G/A	
*qRRE10*	chr07	8,451,115	2.59 × 10^−6^	C/T	
*qRRE11*	chr08	22,766,099	2.45 × 10^−6^	T/C	
*qRRE12*	chr08	28,240,531	2.06 × 10^−6^	T/G	
*qRRE13*	chr09	5,323,335	2.45 × 10^−6^	C/T	
*qRRE14*	chr10	22,657,213	3.96 × 10^−6^	A/T	
*qRRE15*	chr11	8,988,532	3.02 × 10^−6^	C/G	*qZRSDW11-2* *qZRTDW11* [14]; *qZnRL11* [15]
*qRRE16*	chr11	10,391,796	5.15 × 10^−7^	C/T	
*qRRE17*	chr11	27,270,465	1.45 × 10^−6^	C/T	
*qRRE18*	chr12	22,393,995	3.33 × 10^−7^	G/A	
*qRRE19*	chr12	23,423,605	3.60 × 10^−6^	A/G	

## Data Availability

The datasets used during the current study are available from the corresponding author on reasonable request.

## Supplementary Materials

The following supporting information can be downloaded at: https://www.mdpi.com/article/10.3390/plants11223138/s1, Figure S1: GWAS of root elongation in control conditions, a distribution of root elongation of Ting’s core collection grown in control conditions, b: box plots for root elongation under high Zn (120 μM) conditions and without Zn toxicity. The *P*-value of Student’s t-test was shown for comparing root elongation under high and without Zn toxicity, **c**: Manhattan plot of GWAS using root elongation without Zn toxicity. Chromosomes are depicted in different colors. The green line depicts the adjusted significance threshold (*p* = 0.94 × 10^−5^), Figure S2: GWAS of Zn tolerance in high Zn conditions using LMM model, a: Manhattan plot of GWAS using root elongation in high Zn conditions, b: Manhattan plot of GWAS using relative root elongation in high Zn conditions. Chromosomes are depicted in different colors. The horizontal red line depicts the significance threshold (*p* = 2.27 × 10^−7^), and the green line depicts the adjusted significance threshold (*p* = 0.94 × 10^−5^). The black arrows represent the significant loci detected using EMMAX and LMM models. The red arrow represents the same loci as the previously reported under Zn stress, Table S1: root growth of Ting’s core collection in control and Zn toxicity conditions, Table S2: QTL for root elongation of Ting’s core collection in control condition, Table S3: candidate genes associated with Zn tolerance, Table S4: primers used for qRT-PCR.
