# Supplementary material for: Genome-Wide Association Study of Zinc Toxicity Tolerance within a Rice Core Collection (Oryza sativa L.)
Source: Plants (Basel). 2022 Nov 16;11(22):3138. doi: 10.3390/plants11223138 (PMC9693275; doi:10.3390/plants11223138)
Supplement: Supplementary file 1 [file plants-11-03138-s001.zip › plants-1965802-supplementary.pdf]

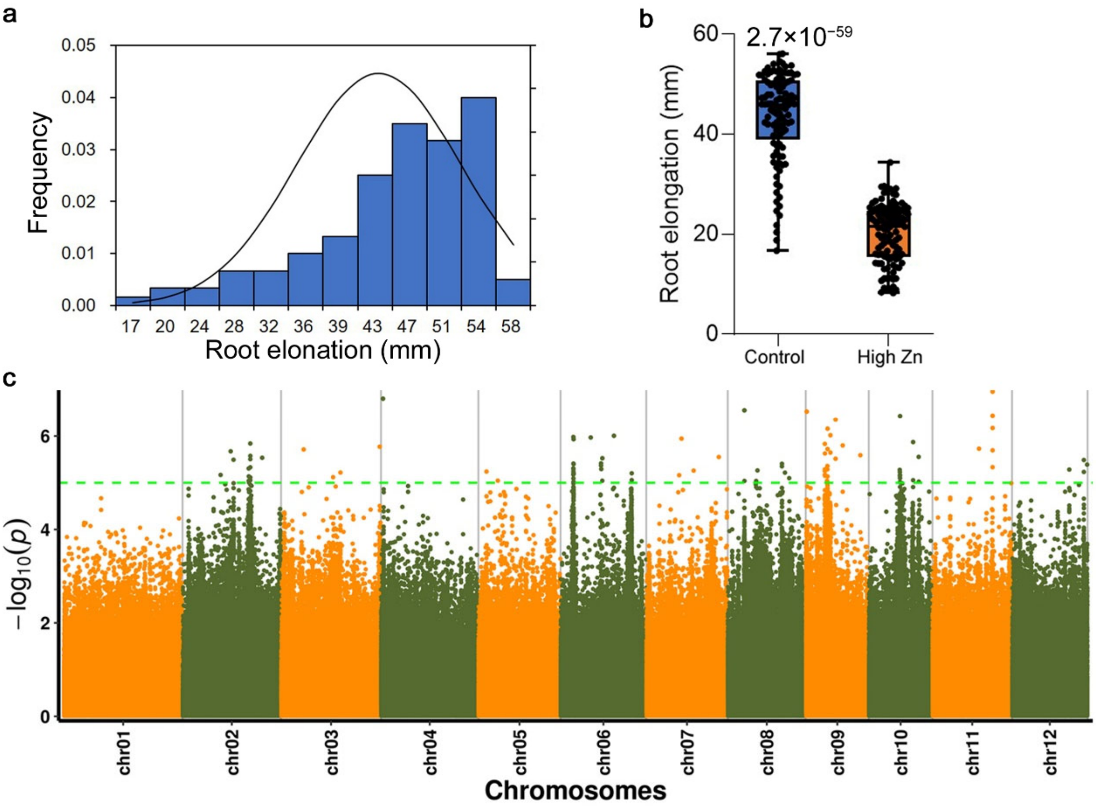

Figure S1. GWAS of root elongation in control conditions. (**a**). Distribution of root elongation of Ting's core collection grown in control conditions; (**b**). Box plots for root elongation under high Zn (120 μM) conditions and without Zn toxicity. The *p*-value of Student's *t*-test was shown for comparing root elongation under high and without Zn toxicity; (**c**). Manhattan plot of GWAS using root elongation without Zn toxicity. Chromosomes are depicted in different colors. The green line depicts the adjusted significant threshold ($p=0.94 \times 10^{-5}$).

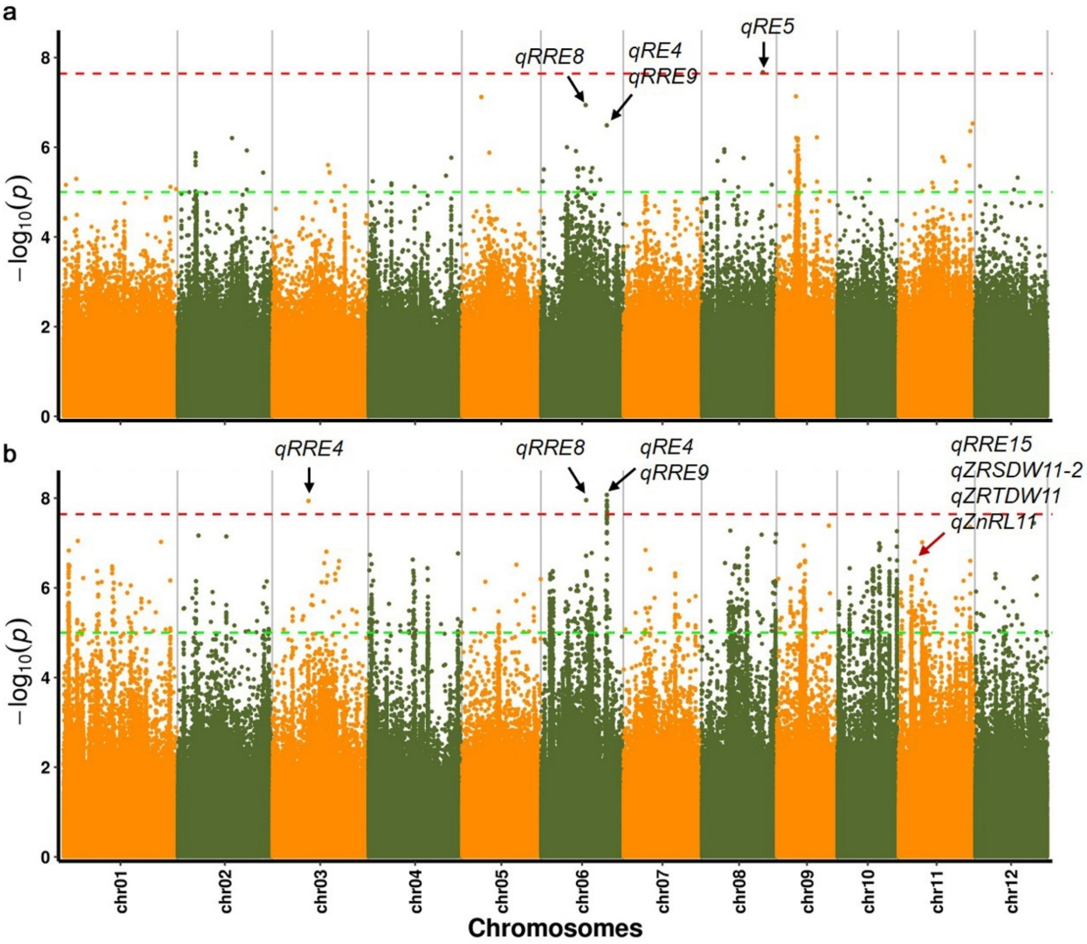

Figure S2. GWAS of Zn tolerance in high Zn conditions using LMM model. (**a**). Manhattan plot of GWAS using root elongation in high Zn conditions; (**b**). Manhattan plot of GWAS using relative root elongation in high Zn conditions. Chromosomes are depicted in different colors. The horizontal red line depicts the significant threshold (p=2.27 × 10^{-7}) and the green line depicts the adjusted significant threshold (p=0.94 × 10^{-5}). The black arrows represent the significant loci detected using EMMAX and LMM models. The red arrow represents the same loci as the previously reported under Zn stress.

Table S1. Root growth of Ting's core collection in control and Zn toxicity conditions.

ID	Variety Name	Root Elongation (mm, control) ±S.D.	Root Elongation (mm, high Zn) ±S.D.	Relative Root elongation (%)	Origin
S001	Yin guang	46.30±2.54	24.67±2.69	53.28±5.82	Japan
S005	Guo zhu	40.89±2.93	17.50±3.41	42.80±8.33	Japan
S007	Mang shui dao	45.87±4.10	21.10±6.94	46.00±9.50	Yangtze River region
S008	Bai mang gao li han dao bai	47.13±3.02	16.06±2.58	34.06±5.47	North China
S010	Bi jie ma wei hong gu	42.40±4.50	18.55±3.24	43.74±7.63	Yunnan-Kweichow Plateau
S011	Ai da tou	23.76±3.15	13.44±1.46	56.57±6.16	Yangtze River region
S012	Gui zao bai he	49.50±4.16	19.80±5.33	40.00±9.10	Yangtze River region
S013	Xiang dao	35.62±4.13	11.19±2.32	31.41±6.50	North China
S014	Zi jin gu	42.53±3.25	21.35±2.32	50.20±5.45	Northeast China
S015	Xiang chuan	38.25±2.12	13.30±1.70	34.77±4.45	Japan
S016	Nagabo	16.71±2.82	13.06±3.19	78.19±10.10	Taiwan
S018	San pai zhong	33.43±5.12	15.38±2.47	45.99±7.40	South China
S019	Kai xuan	50.44±4.24	16.06±5.07	31.84±8.58	Japan
S020	Shi ban zhan	45.53±5.00	21.06±2.51	46.25±5.52	North China
S022	Shen shui wan dao	31.50±6.81	8.75±0.96	27.78±3.04	Yangtze River region
S023	Hong ben dao	50.88±3.26	15.35±4.03	30.17±7.92	Yangtze River region
S024	Duan mang zi jin gu	18.82±5.40	13.19±1.22	70.08±6.50	Northeast China
S025	Bei jing jiang mi	46.08±3.57	22.20±3.78	48.18±8.21	Northeast China
S026	Daeri	35.67±2.91	13.81±4.78	38.73±9.34	Celebes
S027	Jian tou nuo	26.5±5.13	12.38±5.26	46.70±9.37	South China

S028	Long you man dao	54.06±3.77	24.06±4.62	44.50±8.54	Yangtze River region
S029	Kun shan zhu zhou dao	54.24±1.99	25.72±3.43	47.43±6.32	Yangtze River region
S031	Sheng fang da bai gu	42.47±3.79	11.19±2.90	26.34±6.84	North China
S032	Xiao du	30.00±7.07	11.00±1.00	36.67±3.33	Japan
S033	Poetih	39.75±4.80	15.65±2.40	39.36±6.03	Celebes
S034	Tebaro	27.44±4.98	9.27±1.62	33.77±5.92	Sumbawa
S035	Ao hua da gui tou hong	47.94±4.23	17.29±1.65	36.08±3.44	Yangtze River region
S036	Hui bei zi	45.87±2.72	15.08±4.11	32.87±8.97	Yunnan-Kweichow Plateau
S037	Ba shi zi	43.43±3.82	21.33±2.63	49.12±6.07	Yangtze River region
S038	Zao sheng da ye	40.46±4.70	14.15±1.77	34.98±4.38	Japan
S039	Bnlastog	37.85±2.27	9.50±3.57	25.10±7.82	Low latitude region
S040	Nuo mi	50.28±3.37	24.47±4.84	48.67±9.62	North China
S041	Xi chuan huang liu	47.77±3.32	21.53±4.91	45.08±9.00	South China
S042	Hei ju dao	25.62±3.78	8.25±1.77	32.21±6.91	Yangtze River region
S043	Guang fu 1 hao	51.89±2.70	25.44±2.71	49.04±5.21	Taiwan
S044	Zhong qi jia qing	47.33±4.33	15.93±2.71	33.66±5.73	Yangtze River region
S045	III-49-4 xi chuan huang	46.81±4.29	22.47±3.66	52.82±8.61	Taiwan
S046	Xin xian li	47.29±3.79	25.07±4.82	53.00±7.62	Yangtze River region
S047	Da liu tiao dao	48.58±5.74	20.46±3.80	42.12±7.82	Yangtze River region
S048	Bai ke	52.56±3.03	26.00±2.93	49.47±5.58	South China
S049	Chuan chi yi hao	52.61±4.92	23.94±4.49	45.51±8.54	Central China
S050	Tai nong 46	32.71±1.38	9.00±3.16	27.51±6.70	Taiwan
S051	Ba chong sui	47.06±4.96	24.00±3.66	51.00±7.78	Japan
S052	Yun nan bai	21.81±4.20	11.25±1.65	51.58±7.58	Central China
S053	Liao yang ben di 4 hao	41.88±3.90	20.88±2.09	49.84±5.00	Northeast China
S054	You zhan hong	40.89±3.36	8.94±3.73	21.86±7.58	South China
S056	Xian zi zhan	45.82±3.30	17.83±3.24	38.92±7.07	Central China
S057	Da tou meng	44.25±2.44	19.28±3.64	43.57±8.23	Central China

S058	Chi bai gan zhan	50.11±4.44	18.89±2.65	37.69±5.30	Central China
S059	Zeng cheng xiang shan zhan	45.47±2.83	24.61±3.05	54.13±6.71	South China
S060	Cang wu shan he zhan	52.00±3.41	23.22±3.73	44.66±7.18	South China
S061	Da gu zao	52.94±4.26	27.12±2.67	51.22±5.04	South China
S062	Jie yang dong liao zhong	47.94±2.41	26.29±3.87	54.85±8.07	South China
S063	Nan xiong ku gua zao	48.18±7.18	25.38±3.43	52.69±7.12	South China
S064	Chang mang	41.59±5.04	24.53±3.72	58.99±8.94	South China
S065	Mandi	42.25±2.72	23.63±4.22	55.92±6.25	Celebes
S066	Bai gu	29.60±7.92	18.25±3.81	61.66±6.72	South China
S068	Can wu shan he zhan	42.36±4.38	22.06±4.34	52.09±6.89	South China
S069	Zao die zhan gu	52.33±4.06	22.94±4.44	43.84±8.48	Central China
S070	Su zhou zhan	20.44±5.34	8.42±2.50	41.17±6.94	Central China
S072	Luo ding zhan 1	45.44±8.19	21.53±2.21	47.38±4.87	South China
S073	Geng yin 29	46.00±3.64	26.28±3.56	57.13±7.74	South China
S074	Wu ke nuo	44.89±7.19	15.53±3.48	34.60±7.76	South China
S075	Hua bai ke	34.44±6.17	23.60±2.01	68.52±5.84	South China
S076	Guang ye hong mi	51.93±3.20	22.50±7.38	43.32±6.74	South China
S077	Da nuo	43.00±4.68	23.00±2.00	53.49±4.65	South China
S078	Bai xu	28.38±4.68	15.00±3.79	52.85±10.14	South China
S079	Mao he	51.06±2.58	22.63±2.50	44.31±4.90	South China
S080	Xu zai	36.50±8.13	19.71±1.38	54.01±3.78	South China
S083	Chi mao zhan	50.94±2.69	25.00±2.12	49.07±4.16	South China
S084	Hu bei zao	43.47±2.85	21.81±2.64	50.18±6.07	Central China
S087	Dong jun zi	44.50±3.54	26.67±4.16	59.93±9.36	Central China
S091	Hei nuo	56.00±4.21	24.88±2.52	44.43±4.50	Unknown
S093	Gai cao zhan	50.82±2.98	25.00±3.02	49.19±5.94	Central China
S094	Gamal	51.59±2.74	25.31±2.44	49.07±4.73	Unknown
S095	Bu gou wei	40.89±7.18	25.29±2.50	61.84±6.11	South China

S096	Bai ke xi nuo	53.80±2.93	27.93±4.11	51.92±7.65	South China
S097	Ben dao	37.36±3.00	16.25±3.77	43.5±4.64	North China
S098	Ba xian shu	56.12±3.22	26.67±5.41	47.52±6.93	Japan
S099	Guang hong mi dao	43.86±2.63	19.94±3.52	45.48±8.03	Yangtze River region
S100	Wu mang yan guo qing	44.14±4.00	17.00±3.98	38.51±7.65	North China
S101	Chang xu nuo	52.33±4.13	25.88±3.64	49.44±6.96	South China
S103	Bai hua er	54.13±4.18	22.33±2.03	41.26±3.75	South China
S104	Liu chang xian	34.00±9.83	14.2±3.70	41.76±9.72	South China
S105	Bai yin 3	24.69±6.05	10.71±2.76	43.40±9.21	South China
S106	Shui zao huang pi	51.83±3.09	29.61±3.53	57.13±6.82	South China
S107	Yin 2 dong 7	31.53±5.88	11.17±3.69	35.43±7.99	South China
S108	Hou ma	50.56±2.76	23.82±2.58	47.12±5.10	South China
S109	Dong zhu 2 hao	53.57±7.08	34.39±2.30	64.19±4.30	South China
S110	Hong gen da mi	52.78±2.02	19.44±4.32	36.83±8.19	South China
S111	Ben cheng guan yin zhan	46.35±5.11	29.07±3.56	62.26±7.75	Central China
S112	Xi miao gu	52.47±5.24	25.69±2.80	48.96±5.33	South China
S113	186-zao guan yin zhan	50.11±4.54	29.08±4.34	58.04±8.66	Central China
S114	Chang mang hei ma zao	52.39±1.75	19.31±2.09	36.86±3.99	Yunnan
S115	Shui tian zhan gu nuo	49.53±2.65	28.10±4.51	56.73±9.10	South China
S116	Chang han da hua ke	45.11±7.26	14.29±2.52	31.69±5.58	South China
S117	Da he	47.41±3.57	22.53±3.07	47.53±6.47	South China
S118	Zeng cheng hei nuo	49.89±3.31	17.71±4.87	35.51±9.77	South China
S119	Mao he	42.00±7.55	29.50±0.71	70.24±1.68	South China
S121	Die zhi	34.00±6.24	16.00±4.98	47.06±8.75	South China
S122	You zhan	52.25±2.54	28.33±3.93	54.23±7.51	South China
S124	Tong ling hu nan xian	38.00±7.79	23.33±2.29	61.40±6.03	Central China
S125	Xiao mao dao	47.13±4.78	29.18±4.03	61.90±8.56	Central China
S127	Zi xing er qu si du xu	46.00±4.03	24.00±2.51	52.17±5.45	Central China

S128	Chen hui fu dao	46.07±2.06	16.69±5.20	36.23±5.74	Central China
S129	Bai gan zi	41.83±7.33	25.35±5.04	60.6±8.35	Central China
S130	Zao lu wei zhan	53.33±2.54	23.22±4.11	43.54±7.71	Central China
S132	Ta gu zhan	52.06±3.17	22.94±2.65	44.08±5.08	Central China
S133	Mian tiao zhan	36.24±3.42	14.27±2.55	39.37±7.03	Central China

Table S2. QTL for root elongation of Ting's core collection in control condition.

QTL	Chromosome	Position	*p*-value	Allele	Physical interval (bp)
qCRE1	chr02	17,552,474	2.12E-06	G/A	17,446,354–17,645,569
qCRE2	chr02	18,591,797	3.22E-06	A/C	18,495,340–18,662,179
qCRE3	chr02	24,115,867	4.95E-06	C/T	24,021,764–24,201,250
qCRE4	chr02	24,704,915	1.44E-06	T/C	24,645,508–24,786,542
qCRE5	chr02	29,034,587	2.92E-06	T/A	28,935,436–29,120,637

qCRE6	chr03	8,228,307	1.94E-06	G/A	8,130,378–8,328,581
qCRE7	chr03	21,631,477	6.04E-06	A/G	21,539,060–21,731,479
qCRE8	chr03	35,942,382	1.69E-06	C/T	35,840,625–36,043,821
qCRE9	chr04	728,686	1.55E-07	A/T	639,804–798,794
qCRE10	chr05	2,953,058	5.76E-06	C/T	2,852,853–3,056,996
qCRE11	chr06	4,675,238	1.05E-06	A/C	4,577,367-4,764,420
qCRE12	chr06	10,991,829	1.07E-06	T/G	10,891,500–11,094,202
qCRE13	chr06	15,233,394	2.99E-06	A/G	15,189,242–15,254,487
qCRE14	chr06	19,455,509	9.87E-07	A/T	19,357,232–19,553,885
qCRE15	chr06	25,944,857	6.40E-08	C/T	2,5867,747–26,040,374
qCRE16	chr07	12,831,029	1.14E-06	G/A	12,747,743–12,930,673
qCRE17	chr07	17,266,036	5.49E-06	T/C	17,186,673–17,285,379
qCRE18	chr07	26,443,312	2.81E-06	G/A	26,340,582–26,545,180
qCRE19	chr08	6,072,098	2.75E-07	C/G	5,982,519–6,175,794
qCRE20	chr08	10,838,488	5.45E-06	T/C	10,759,593–10,945,133
qCRE21	chr08	19,756,555	3.92E-06	A/G	19,657,493–19,854,635
qCRE22	chr08	20,851,712	6.08E-06	A/T	20,761,382–20,947,979
qCRE23	chr08	22,400,585	7.95E-06	T/C	22,334,606–22,497,785
qCRE24	chr09	346,485	2.93E-07	G/C	244,493–451,698
qCRE25	chr09	6,954,135	1.47E-06	G/A	6,873,236–7,038,397
qCRE26	chr09	7,960,191	6.95E-07	T/G	7,871,403–8,046,886
qCRE27	chr09	10,866,545	4.41E-07	C/T	10,763,252–10,969,484
qCRE28	chr09	13,467,437	1.59E-06	T/C	13,376,227–13,575,139
qCRE29	chr09	19,898,066	2.57E-06	G/A	19,796,232–19,999,811
qCRE30	chr10	11,413,443	3.66E-07	G/A	11,314,672–11,464,119
qCRE31	chr10	16,156,147	1.35E-06	G/A	16,084,478–16,243,154
qCRE32	chr10	18,183,013	2.79E-06	A/T	18,085,539–18,281,584
qCRE33	chr11	16,978,564	1.87E-06	A/G	16,885,485–17,066,284

qCRE34	chr11	21,930,706	1.10E-07	A/T	21,839,186–22,031,935
qCRE35	chr12	20,866,616	5.22E-06	A/T	20,781,339–20,903,625
qCRE36	chr12	26,176,316	3.26E-06	G/A	26,081,664–26,270,792
qCRE37	chr12	27,362,251	4.09E-06	C/T	27,263,777–27,464,059

CRE: root elongation in control conditions.

Table S3. Candidate genes associated with Zn tolerance.

QTL	Gene ID	Annotation
	Os02g0650300	OsYSL15
	Os02g0650500	protein kinase domain containing protein
	Os02g0650800	CHR740
	Os02g0650900	glutamate dehydrogenase protein
	Os02g0651000	expressed protein
	Os02g0651200	expressed protein
	Os02g0651300	uncharacterized protein
	Os02g0651500	expressed protein
qRE2	*Os02g0651900*	--
	Os02g0652000	cyclin-P4-1-like
	Os02g0652100	OsWRKY34
	Os02g0652300	ELMO/CED-12 family protein
	Os02g0652550	MSP domain containing protein
	Os02g0652575	Hypothetical gene
	Os02g0652600	50S ribosomal protein L19
	Os02g0652800	Transporter
	Os02g0653000	--

	Os02g0653200	plastocyanin-like domain containing protein
	Os02g0653300	Conserved hypothetical protein
	Os02g0653400	transferase family protein
	Os02g0653800	ras-related protein
	Os02g0653850	Hypothetical gene
	Os02g0653900	triacylglycerol lipase like protein
	Os02g0654000	enoyl-CoA hydratase
	Os02g0654100	enoyl-CoA hydratase
qRE5	*Os08g0470700*	bifunctional monodehydroascorbate reductase and carbonic anhydrasenectarin-3 precursor
	Os08g0471000	OsHsfB4a
	Os08g0471150	Hypothetical protein
	Os08g0471300	Similar to Methyl-CpG binding protein-like
	Os08g0471800	remorin C-terminal domain containing protein
	Os08g0471850	Hypothetical protein
	Os08g0471900	zinc finger family protein
	Os08g0472000	OsbZIP66
	Os08g0472400	expressed protein
	Os08g0472600	FucT
	Os08g0472800	OsABA8ox2
	Os08g0473200	Hypothetical protein
qRRE1	*Os01g0199300*	lachrymatory factor synthase
	Os01g0199400	Hydrolase
	Os01g0199550	--
	Os01g0199700	expressed protein
	Os01g0199900	phosphoribosylaminoimidazole carboxylase
	Os01g0199950	Hypothetical gene

	Os01g0200000	autophagy-related protein 3
	Os01g0200150	Hypothetical gene
	Os01g0200200	expressed protein
	Os01g0200300	OSHB5
	Os01g0200350	Hypothetical protein
	Os01g0200400	expressed protein
	Os01g0200500	MPPN domain containing protein
	Os01g0200600	AP2 domain containing protein
	Os01g0200700	OsMTI-3a
	Os01g0200950	--
	Os01g0201000	protein kinase family protein
	Os01g0201100	xylosyltransferase
	Os01g0201200	protein kinase domain containing protein
	Os01g0201250	HD1
	Os01g0201275	expressed protein
	Os01g0201300	expressed protein
	Os01g0201400	RALFL17
	Os01g0201500	plastocyanin-like domain containing protein
	Os01g0201600	OsGAE1
	Os01g0201700	OSMADS3
	Os01g0201750	--
	Os01g0201800	expressed protein
	Os01g0201850	Hypothetical conserved gene
qRRE4	*Os03g0356470*	expressed protein
	Os03g0356477	--
	Os03g0356484	tetratricopeptide repeat containing protein
	Os03g0356498	rhythmically expressed gene 2 protein

	Os03g0356526	expressed protein
	Os03g0356540	CXXC1
	Os03g0356582	phosphatidylinositol-4-phosphate 5-kinase
	Os03g0356596	phosphatidylinositol-4-phosphate 5-kinase
	Os03g0356638	pleckstrin homology domain-containing protein
	Os03g0356652	TRAF-type zinc finger domain-containing protein 1
	Os03g0356700	VLN2
	Os03g0356801	--
	Os03g0356900	OsIPT2
	Os03g0357200	--
	Os03g0357400	Hypothetical conserved gene
	Os03g0357500	Protease inhibitor
	Os03g0358000	PWWP domain containing protein
qRRE8	*Os06g0493600*	OsPHO1;3
	Os06g0493700	Pi homeostasis
	Os06g0493800	Conserved hypothetical protein
	Os06g0493801	pentatricopeptide
	Os06g0493900	Conserved hypothetical protein
	Os06g0494000	expressed protein
	Os06g0494100	Pid2
	Os06g0494250	Hypothetical protein
	Os06g0494400	MATE efflux family protein
	Os06g0494701	retrotransposon protein
	Os06g0495100	MATE domain containing protein
	Os06g0495500	MATE efflux family protein
	Os06g0495632	Similar to F-box domain containing protein
	Os06g0495700	PSP domain containing protein

	Os06g0495800	DUF617 domain containing protein
qRE4/qRRE9	*Os06g0621600*	CC-NBS-LRR protein
	Os06g0621800	retrotransposon protein
	Os06g0621900	expansin precursor
	Os06g0621950	Hypothetical protein
	Os06g0622000	CW-type Zinc Finger
	Os06g0622300	OsARID3
	Os06g0622400	transposon protein
	Os06g0622500	tetratricopeptide repeat domain containing protein
	Os06g0622550	retrotransposon protein
	Os06g0622700	OsbZIP50
	Os06g0622800	guanine nucleotide exchange family protein
	Os06g0622900	RNA recognition motif containing protein
	Os06g0623050	guanine nucleotide exchange family protein
	Os06g0623200	dihydroflavonol-4-reductase
	Os06g0623300	reductase
	Os06g0623600	reductase
	Os06g0623700	TGW6
	Os06g0624000	expressed protein
	Os06g0624100	OsSub51
	Os06g0624200	hypothetical protein
	Os06g0624700	expressed protein
	Os06g0624750	Hypothetical protein
qRRE12	*Os08g0562100*	lactate/malate dehydrogenase
	Os08g0562200	OsNTL5
	Os08g0562300	C2H2 zinc finger protein
	Os08g0562500	BAHD acyltransferase-like protein gen

	Os08g0562600	C2 domain containing protein
	Os08g0562700	aminopeptidase
	Os08g0562750	--
	Os08g0562800	MATE efflux family protein
	Os08g0562866	Hypothetical protein
	Os08g0563200	DNA binding protein
	Os08g0563300	SNARE domain containing protein
	Os08g0563400	DUF260 domain containing protein
	Os08g0563450	Hypothetical protein
	Os08g0563500	C3HC4 type domain containing protein
	Os08g0564000	OsPT6
	Os08g0564100	ABC transporter
	Os08g0564200	Hypothetical protein
	Os08g0564300	SD8
	Os08g0564400	Hypothetical protein
	Os08g0564500	zinc finger protein 593
	Os08g0564700	protein Kinase-like protein TMKL1 precursor
	Os08g0564750	expressed protein
	Os08g0564800	expressed protein
	Os08g0564900	Conserved hypothetical protein
	Os08g0565200	AP2 domain containing protein
	Os08g0565450	Similar to H0107B07.4 protein
qRRE15	*Os11g0264900*	expressed protein
	Os11g0264950	--
	Os11g0265000	uridine/cytidine kinase-like 1
	Os11g0265100	Hypothetical protein
	Os11g0265200	expressed protein

Os11g0265400	oxidoreductase
Os11g0265450	--
Os11g0265500	uncharacterized protein
Os11g0263000	NBS-LRR disease resistance protein
Os11g0263466	--
Os11g0264000	laccase precursor protein
Os11g0264200	F-box domain containing protein
Os11g0264300	NIN
Os11g0264500	expressed protein
Os11g0264600	expressed protein
Os11g0264700	expressed protein

Table S4. Primers used for qRT-PCR

Primer name	Sequence 5'–3'
OsUBQ1-F	GCTCCGTGGCGGTATCAT
OsUBQ1-R	CGGCAGTTGACAGCCCTAG
Os01g0200700-F	TATTTGCTTGCTTTGTGCGT
Os01g0200700-R	TTCGGTTGCTTCGTGACTTC
Os06g0493600-F	AACCCTTGGCTCCGTAATGA
Os06g0493600-R	ACCTCAAGAGCTGCCAAGAT
Os06g0621600-F	TTGGCCATGACAAGTTTCGG
Os06g0621600-R	AGCTGGAGATACGCCATTGA

Os06g0621900-F	CATCTTCAGGTACGGGAGGT
Os06g0621900-R	ATCTCGAAGTGCTCCTTGGG
Os06g0622300-F	CATTGGTTCCTGGCCTTCTG
Os06g0622300-R	ATGGTGCACGGACAAATAGC
Os06g0622700-F	GCGATGATCCCATGAGCAAG
Os06g0622700-R	CCGACAGGCCTATCCTTCAA
Os11g0263000-F	AGAGTGGGATGTTCAGCGAT
Os11g0263000-R	CCAGGTTCTTTAGGGAGCCA
Os11g0264200-F	ATGGTCTGCGATTGGATTGC
Os11g0264200-R	CCTGCAAACCTTGGTCACAA
